# Frequency-specific gyro-sulcal functional differentiation in preterm neonates: Sulcal dominance and gene expression correlates

**DOI:** 10.1016/j.dcn.2026.101829

**Published:** 2026-09-29

**Authors:** Wei Mao, Xun Ou, Wenxuan Huang, Xuewei Jin, Keith Kendrick, Xi Jiang

**Affiliations:** The Clinical Hospital of Chengdu Brain Science Institute, MOE-K Lab for NeuroInformation, Brain‑Apparatus Communication Institute, School of Life Science and Technology, University of Electronic Science and Technology of China, Chengdu, 611731, China

**Keywords:** Preterm neonates, Gyri and sulci, Frequency-specific functional differentiation, Gene expression

## Abstract

Preterm birth affects up to 10.6% of neonates and increases neurodevelopmental risks, yet functional brain alterations remain poorly understood. Using resting-state fMRI data from 437 neonates and a one-dimensional convolutional neural network, this study investigated frequency-specific functional differences between gyri and sulci across three groups: preterm-born neonates scanned at preterm age, preterm-born neonates scanned at term-equivalent age, and term-born neonates scanned at term age. Associations with gene expression were examined. Group differences in the model-derived frequency characteristics were observed predominantly in the frontal lobe across all frequency bands, and in the temporal and parietal lobes at high frequencies. These differences were more pronounced for sulcal than gyral characteristics, and sulcal dominance was more spatially extensive in neonates scanned at term-equivalent age than in preterm neonates scanned before term-equivalent age. Furthermore, model-derived gyro-sulcal frequency characteristics were significantly associated with regional cortical gene-expression patterns. This study bridges frequency-specific brain imaging with cortical gene expression, highlighting the prominent contribution of sulcal-related frequency characteristics to the observed group differences. These findings reveal regional associations between gyro-sulcal frequency characteristics and gene expression and suggest their potential relevance for early risk assessment, which requires further validation.

## 1 Introduction

It is reported that approximately 15 million infants are born preterm globally each year (Chawanpaiboon et al., 2019). Complications arising from preterm birth are major contributors to mortality in children under five years of age (Liu et al., 2016). Preterm birth disrupts typical brain developmental trajectories, which may lead to alterations in neural activity and functional brain development. Surviving preterm neonates are vulnerable to a wide spectrum of short- and long-term neurodevelopmental complications (Bitner-Glindzicz et al., 2014, Blencowe et al., 2013; Demeši Drljan et al., 2016; Erbek et al., 2009; Franz et al., 2018; Hee Chung et al., 2020; Kuzniewicz et al., 2014; Lima et al., 2006; Moreno-Plata et al., 2011; Mwaniki et al., 2012; Platt, 2014; Saigal and Doyle, 2008). Resting-state functional network organization has also been associated with individual differences in neurodevelopment-related traits (Hagan et al., 2025). While previous studies have documented alterations in functional connectivity in preterm compared with term-born infants (Eyre et al., 2021, França et al., 2024, Hamouda et al., 2026, Mao et al., 2026), a more comprehensive characterization of their functional neural activity profiles remains essential to support earlier diagnosis and inform clinical management. However, few studies have focused on the frequency-specific differentiation of functional signals between gyri and sulci in preterm neonates, limiting our understanding of the early neural mechanisms underlying their neurodevelopmental abnormalities.

Cortical gyrification is a crucial neurodevelopmental milestone, and the resulting gyri and sulci are the most prominent morphological features of the mammalian cerebral cortex (Deng et al., 2014, Jiang et al., 2015, Liu et al., 2019). These structures differ markedly in neuronal distribution, gene expression, and various structural and functional attributes (de Juan Romero et al., 2015, Deng et al., 2014, Hilgetag and Barbas, 2005, Jiang et al., 2015, Jiang et al., 2018, Nie et al., 2012, Rakic, 2009, Wang et al., 2023a). Notably, functional MRI (fMRI)-based studies have revealed distinct signal properties between these regions. For instance, BOLD signals in gyral regions exhibit less diverse temporal patterns than those in sulcal regions (Yang et al., 2019). Studies utilizing both resting-state (rs-fMRI) and task-based (t-fMRI) have consistently shown that BOLD signals in gyri are of lower frequency than those in sulci, as observed at both whole-brain and regional scales (Liu et al., 2017, Yang et al., 2019). A growing body of evidence suggests that gyri and sulci play specialized roles in brain function: gyri may serve as global information exchange centers, interacting directly with distant gyri and neighboring sulci, whereas sulci act as local information processing units, primarily engaging with neighboring regions and interacting with distant regions indirectly via gyral associations (de Juan Romero et al., 2015, Nie et al., 2012, Zhang et al., 2019). Given that gyrification is reported to be altered in preterm neonates (Hedderich et al., 2019), such abnormalities may further lead to altered functional differentiation between gyri and sulci. Thus, investigating potential alterations in gyro-sulcal functional differentiation in preterm newborns could provide a valuable framework for understanding the neural mechanisms underlying the cognitive and psychiatric challenges associated with prematurity.

Deep learning models have been widely used in medical imaging in recent years (Chen et al., 2024, Jiang et al., 2024, Jiang et al., 2023b, Shen et al., 2017, Yan et al., 2022, Zhao, 2025). Many recent studies based on human and primate brains have successfully classified fMRI signals in gyri and sulci using one-dimensional convolutional neural networks (1D-CNNs) and have further demonstrated differences in their frequency characteristics by analyzing the spectra of the model’s learned convolutional filters (Liu et al., 2019, Wang et al., 2024, Zhang et al., 2019). Because the newborn brain develops rapidly and differs from that of adults, these 1D-CNN studies inspired us to examine model-derived frequency characteristics related to gyro-sulcal functional differentiation in preterm and term neonates.

Therefore, this study utilizes multi-modal MRI data from the Developing Human Connectome Project (dHCP) to investigate frequency-specific functional differences between gyri and sulci in preterm versus term neonates. We hypothesized that preterm neonates would exhibit altered gyro-sulcal frequency differentiation compared to term-born neonates, primarily driven by sulcal regions and correlated with cortical gene expression. A 1D-CNN model was employed to test this hypothesis across three neonatal groups: preterm neonates scanned immediately after birth, preterm neonates scanned at term-equivalent age, and term neonates. Furthermore, we explored the associations between gyro-sulcal frequency characteristics and cortical gene expression profiles. By integrating model-derived frequency characteristics with gene-expression data, this work provides a framework for investigating prematurity-related brain alterations and their regional gene-expression associations.

## 2 Materials and methods

### 2.1 Dataset and preprocessing

We used 437 neonatal subjects with T2w-MRI and rs-fMRI data from the Developing Human Connectome Project (dHCP, http://www.developingconnectome.org/) with authorization. The study was approved by the National Research Ethics Service West London committee, and written informed consent was obtained from participating families prior to imaging. Based on gestational age (GA) at birth and postmenstrual age (PMA) at scan, all subjects were divided into three groups: 86 preterm-born neonates scanned at preterm age (PP; GA≤37 weeks and PMA≤37 weeks), 80 preterm-born neonates scanned at term-equivalent age (PT; GA ≤37 weeks and PMA>37 weeks), and 271 term-born neonates scanned at term age (TT; GA >37 weeks and PMA >37 weeks). The distributions of GA and PMA are presented in Table 1.

**Table 1:** Distribution of GA at birth and PMA at scan in the three neonatal groups.

Groups	N	GA at birth (weeks)	PMA at scan (weeks)
PP	86	33.66±2.96	35.47±1.58
PT	80	32.63±3.71	40.90±1.91
TT	271	40.11±1.03	41.17±1.55

**Note:** Values are presented as mean ± SD. GA, gestational age; PMA, postmenstrual age; PP, preterm-born neonates scanned at preterm age; PT, preterm-born neonates scanned at term-equivalent age; TT, term-born neonates scanned at term age.

The neonatal brain MRI scans were acquired during natural sleep using a 3 T Philips Achieva scanner. The scanning parameters for T2-weighted MRI (T2w-MRI) were as follows: spatial resolution = 0.8 mm × 0.8 mm × 1.6 mm, TR = 12 s, TE = 156 ms, axial SENSE factor = 2.11, and sagittal SENSE factor = 2.58. The preprocessing pipeline for T2w-MRI, including skull removal, tissue segmentation, and surface reconstruction, is described in detail in the dHCP T2w-MRI pipeline (Makropoulos et al., 2018). The rs-fMRI parameters were as follows: spatial resolution = 2.15 mm × 2.15 mm × 2.15 mm, TR = 0.392 s, TE = 38 ms, with 2300 volumes. Detailed preprocessing for rs-fMRI is described in the dHCP functional pipeline (Fitzgibbon et al., 2020). Subsequently, rs-fMRI volumes were registered to T2w-MRI using FSL-FLIRT (Jenkinson et al., 2002, Jenkinson and Smith, 2001).

We adopted publicly available gene expression data from the PsychEncode Project (http://development.psychencode.org/) with authorization (Li et al., 2018). The data were collected with consent from parents or legal guardians and were approved by the local institutional review boards. Data handling followed the ethical guidelines and regulations set by the NIH and the WMA Declaration of Helsinki. Gene expression data of mRNA measured in RPKM (reads per kilobase per million mapped reads) are selected from 19 prenatal samples, including 16 preterm samples ranging from 12 postconceptional weeks (PCWs) to 37 PCWs, as well as 3 term samples at 4 months. We then generated one preterm and one term averaged gene expression atlas, each with 11 regions of interest (ROIs) per hemisphere. Notably, the right hemisphere of the term atlas had six ROIs with gene expression due to the limited number of samples, and the remaining five ROIs were mirrored from the left hemisphere. All 22 ROIs (11 ×2) were then mapped to the cortical surface space of the dHCP data (Ball et al., 2020).

### 2.2 Definition of brain ROIs, Gyri, and Sulci

We employed the official brain atlas provided by dHCP for MRI data (Gousias et al., 2012). First, the brain atlas was registered to the T2w-MRI volume space and mapped onto the cortical surface, where the white matter surface was divided into 32 brain regions. Within each region, cortical surface vertices were further classified according to their curvature values r (Jiang et al., 2021). Vertices with $r>r_{\mathrm{thr}}$ were classified as gyral vertices, whereas those with $r<-r_{\mathrm{thr}}$ were classified as sulcal vertices, where $r_{\mathrm{thr}}=0.15$ (Fig. 1a). Each of the 32 cortical regions was therefore divided into a gyral and a sulcal subregion, resulting in 64 cortical ROIs. The BOLD signals from rs-fMRI were mapped to cortical surface vertices using nearest- neighbor interpolation (Fig. 1b). The correspondence between the 22 ROIs from the gene expression atlas and the 64 ROIs from the MRI data atlas was established within the same dHCP space.

**Fig. 1:**
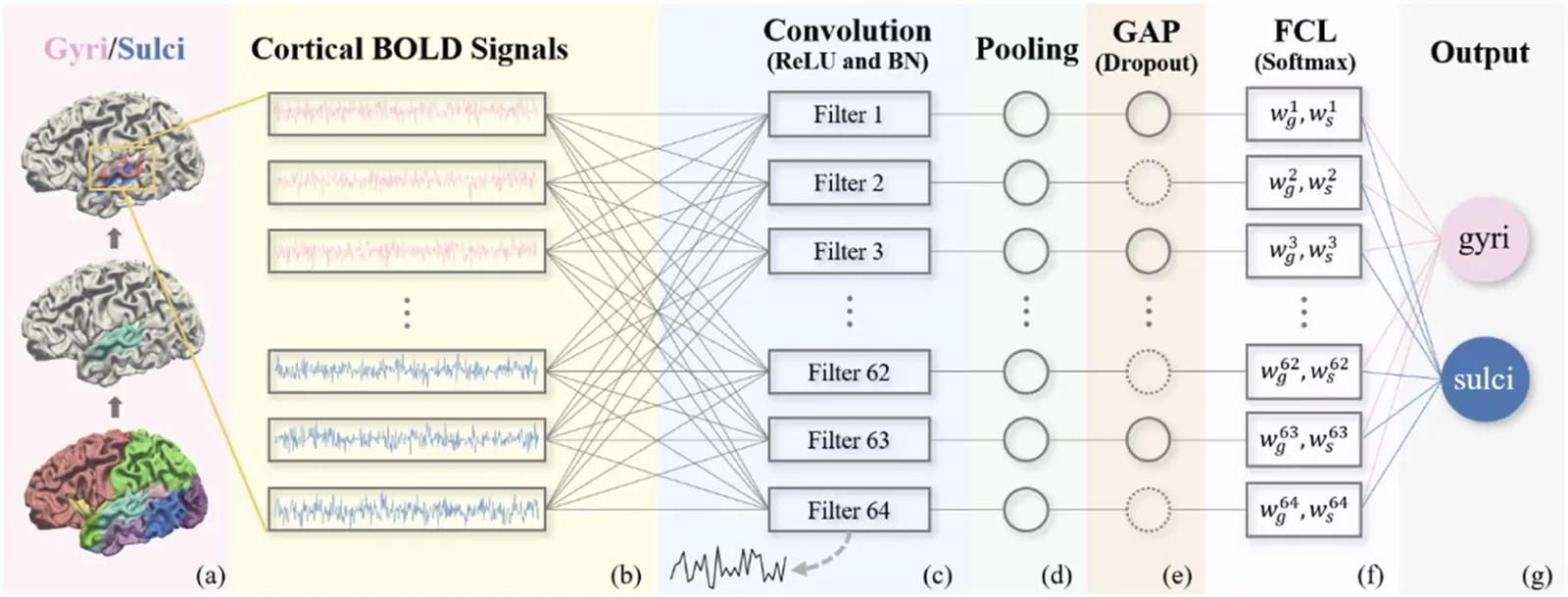
Flowchart of the computational framework. (a) Each brain region is divided into gyrus and sulcus based on cortical curvature; (b) Extraction of fMRI BOLD signals from gyri and sulci; (c)-(g) The structure of the 1D-CNN model, with a total of 64 channels in the middle layer. The convolutional layer, max-pooling layer, global average pooling layer, fully connected layer, and outputs are shown from (c) to (g), respectively.

### 2.3 Overview of the computational framework

The 1D-CNN model can effectively classify fMRI signals from gyral and sulcal regions in both human and non-human primate brains, and the spectra of its learned convolutional filters can be analyzed to characterize the frequency-related information used by the model for this classification (Liu et al., 2019, Wang et al., 2024, Zhang et al., 2019). We developed a 1D-CNN model to classify rs-fMRI BOLD signals from the 64 sulcal and gyral cortical regions across the three neonatal groups. Through this process, the learned convolutional filters captured frequency-related features used by the model to distinguish gyral from sulcal BOLD signals across the three neonatal groups.

The architecture of the 1D-CNN is shown in Fig. 1 and includes a convolutional layer (Fig. 1c), a max-pooling layer (Fig. 1d), a global average pooling (GAP) layer (Fig. 1e), and a fully connected layer (FCL, Fig. 1f). The convolutional layer consists of 64 convolutional filters (i.e., kernels), each followed by batch normalization (BN) and a rectified linear unit (ReLU) activation function. This is followed by a pooling operation and a dropout layer to reduce overfitting. The GAP layer reduces the number of parameters and extracts global features. Finally, the FCL layer applies a softmax function to generate the output, classifying the input into two categories: gyri and sulci (Fig. 1g).

### 2.4 Definition of gyral and sulcal filters

Since convolutional filters can effectively capture either gyrus-specific or sulcus-specific feature information for BOLD signal classification (Liu et al., 2019, Zhang et al., 2019), we classified filters capturing gyral or sulcal feature information as gyral filters or sulcal filters, respectively. We then used the spectra of these learned gyral and sulcal filters to infer frequency-specific gyro-sulcal functional differentiation and further compared these model-derived characteristics among the three neonatal groups.

According to previous studies, each convolutional channel can be associated with a specific output class based on the weights of the corresponding FCL connections (Liu et al., 2019, Zhang et al., 2019). For channel i, $w_{g}^{i}$ and $w_{s}^{i}$ denote the FCL weights connecting the output of that channel to gyral and sulcal output units, respectively. The convolutional filter for channel i was classified according to the signs of these two weights, as defined in Eq. (1):

(1) $${\mathrm{Filter}}^{i}\;\mathrm{Type}=\left\{\begin{array}{c} \mathrm{Gyral},w_{g}^{i}\geq 0\;\mathrm{and}\;w_{s}^{i}\leq 0,\\ \mathrm{Sulcal},w_{g}^{i}\leq 0\;\mathrm{and}\;w_{s}^{i}\geq 0\\ \mathrm{Uncertain},\mathrm{otherwise}. \end{array}\right),i=1,2,\ldots ,64$$

Specifically, a filter was classified as gyral when $w_{g}^{i}\geq 0$ and $w_{s}^{i}\leq 0$, indicating a non-negative contribution to the gyral output and a non-positive contribution to the sulcal output. Conversely, a filter was classified as sulcal when $w_{g}^{i}\leq 0$ and $w_{s}^{i}\geq 0$. Filters that did not satisfy either condition were classified as uncertain.

### 2.5 Measurement of frequency characteristics

The convolutional filter in each of the 64 channels was classified into three types: gyral, sulcal, and uncertain, according to Eq. (1). Only filters classified as gyral or sulcal were included in the subsequent frequency-characteristic analysis. For each gyral or sulcal filter, we extracted the weight parameter sequence $w\left(t\right)$ and calculated its Fourier transform sequence $W\left(f\right)$. The gyral and sulcal functional signals of a brain region were input into the 1D-CNN model, and the mean Fourier transform sequences $\bar{W_{g}}(f)$ of all gyral filters and $\bar{W_{s}}(f)$ of all sulcal filters were calculated as model-derived gyral and sulcal frequency characteristics, respectively. These characteristics reflect the frequency-related information encoded by the learned filters for gyral–sulcal classification, rather than direct spectral or power estimates of the original BOLD signals. The difference $\bar{W_{g}}\left(f\right)-\bar{W_{s}}(f)$ was defined as the model-derived gyro-sulcal differentiation characteristic for that brain region. If $\bar{W_{g}}\left(f\right)-\bar{W_{s}}(f)$ was significantly greater than 0 after a *t*-test with Bonferroni correction, the region was defined as gyri-dominated in terms of the model-derived frequency characteristics; conversely, if it was significantly less than 0, the region was defined as sulci-dominated. Here, dominance refers to the relative magnitude of the model-derived filter-spectrum characteristics and does not indicate greater BOLD power or neural activity.

Furthermore, we calculated pairwise dissimilarities in frequency characteristics between neonatal groups across brain regions. Taking the pairwise dissimilarity in model-derived gyral frequency characteristics as an example, $\bar{W_{\mathrm{ga}}}(f)$ and $\bar{W_{\mathrm{gb}}}(f)$ represent the model-derived gyral frequency-characteristic sequences for the same brain region in two neonatal groups. The Euclidean distance between these two sequences quantifies the degree of dissimilarity between the gyral frequency characteristic patterns of the two groups in that brain region:

(2) $${\mathrm{Dissimilarity}}_{a,b}=d\left(\overset{®}{W_{\mathrm{ga}}}\left(f\right),\overset{®}{W_{\mathrm{gb}}}\left(f\right)\right)$$

where d(∙) denotes the Euclidean distance operator. Euclidean distance was used as a quantitative, non-directional measure of pattern dissimilarity. Pairwise dissimilarities in the model-derived sulcal frequency characteristics and model-derived gyro-sulcal differentiation characteristics were calculated using $\bar{W_{s}}(f)$ and $\bar{W_{g}}\left(f\right)-\bar{W_{s}}(f)$, respectively.

In addition, to further examine the model-derived frequency characteristics across different frequency bands, we referred to previous studies (Liu et al., 2019, Zhang et al., 2019) and divided the frequency characteristic sequences into six commonly used frequency bands: slow1 (0.607–0.694 Hz), slow2 (0.223–0.607 Hz), slow3 (0.082–0.223 Hz), slow4 (0.030–0.082 Hz), slow5 (0.012–0.030 Hz), and slow6 (0.007–0.012 Hz). This division allowed for analysis similar to that conducted across the full frequency band.

### 2.6 Model parameters and training scheme

To reduce overfitting and evaluate the generalizability of gyral–sulcal classification performance across independent data partitions, we used a five-fold cross-validation approach. Due to the high similarity of neighboring signals at cortical locations and to avoid label leakage, all BOLD signals from the same ROI of the same subject were assigned entirely to either the training set or the test set, rather than being split randomly.

In the 1D-CNN, we used 64 convolutional filters, each of length 32. The batch size was set to 1024, and the dropout rate was 0.2. During training and testing, we used 60 epochs with a learning rate of 0.01. The Adam optimizer and CosineDelayLRScheduler were used for learning rate adjustment, with 5 epochs of warm-up to avoid overfitting. The loss function was the cross-entropy loss function.

### 2.7 Association between gene expression and model-derived frequency characteristics

Previous studies have shown a strong relationship between brain function and gene expression (Ball et al., 2020), and that altered gene expression during development caused by preterm birth may lead to cognitive and neuropsychiatric disorders. Therefore, we further investigated group differences in the associations between gene expression and model-derived frequency characteristics across the three neonatal groups. For a brain region n whose signal frequency characteristics $\overset{®}{{\mathrm{FT}}_{n}}$ can be expressed as the model-derived gyral frequency characteristics $\overset{®}{W_{g}}\left(f\right)$, the model-derived sulcal frequency characteristics $\overset{®}{W_{s}}\left(f\right)$, or the model-derived gyro-sulcal differentiation characteristics $\overset{®}{W_{g}}\left(f\right)-\overset{®}{W_{s}}\left(f\right)$, the correlation between the sequence of model-derived frequency characteristics $\overset{®}{\mathrm{FT}}=\{\overset{®}{{\mathrm{FT}}_{1}},\overset{®}{{\mathrm{FT}}_{2}},\cdots ,\overset{®}{{\mathrm{FT}}_{N}}\}$ of the N brain regions and the sequence of gene expression $G\in R^{N}$ was calculated as follows:

(3) $$\rho_{\mathrm{FT}-\mathrm{gene}}=\mathrm{SCC}\left(\overset{®}{\mathrm{FT}},G\right)$$

where $\rho_{\mathrm{FT}-\mathrm{gene}}$ is the correlation between frequency characteristics and gene expression, and $\mathrm{SCC}\left(∙\right)$ is the Pearson correlation coefficient function. The sign and absolute magnitude of Pearson’s correlation coefficient r indicate the direction and strength of the regional imaging–transcriptomic association, respectively.

## 3 Results

### 3.1 Classification of gyral and sulcal BOLD signals

The classification accuracy of gyral and sulcal signals across cortical regions in the three neonatal groups is shown in Fig. 2a. Classification accuracy reflects the model’s ability to distinguish gyral from sulcal BOLD signal patterns. Higher accuracy indicates that the two signal classes were more readily separable by the model and may provide indirect evidence of greater gyro-sulcal functional differentiation. Accordingly, the model achieved the greatest separation of gyral and sulcal signal patterns in temporal regions and the lowest separation in occipital regions. Fig. 2b shows the dominant relationships of gyral and sulcal signals across brain regions at the full-band level, after a *t*-test with Bonferroni correction. In the PP group, most cortical regions were dominated by gyri, with sulcal dominance restricted to limited areas primarily in the temporal and occipital regions. In the PT group, sulcal dominance was observed across more temporal and parietal regions than in the PP group, while gyral dominance was mainly observed in frontal and occipital regions. In the TT group, sulcal dominance was distributed across a broad range of cortical areas, including large portions of the temporal, parietal, and occipital cortices, whereas gyral dominance was largely confined to frontal regions. Taken together, the three cross-sectional groups showed different spatial distributions of gyral and sulcal dominance. Gyral dominance was most extensive in the PP group, whereas sulcal dominance covered broader cortical areas in the PT and TT groups, with the broadest distribution observed in the TT group.

**Fig. 2:**
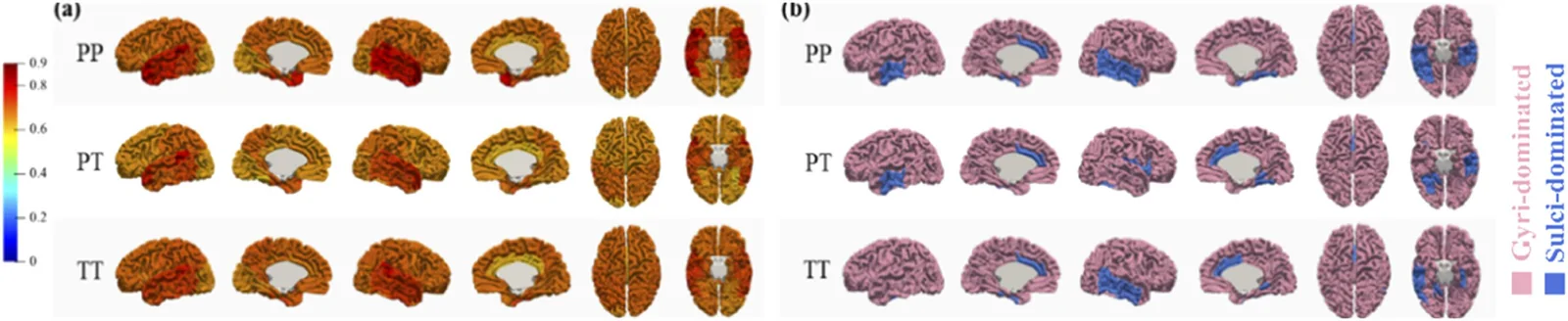
**Gyro-sulcal classification accuracy across brain regions among neonatal groups.** PP, preterm-born neonates scanned at preterm age; PT, preterm-born neonates scanned at term-equivalent age; TT, term-born neonates scanned at term age. (a) The color bar indicates classification accuracy. (b) Pink indicates brain regions dominated by gyri (i.e., gyral characteristics significantly greater than sulcal characteristics), and blue indicates regions dominated by sulci.

### 3.2 Band-specific dominance in model-derived frequency characteristics

We divided the model-derived gyral and sulcal frequency-characteristic sequences into six commonly used frequency bands (slow1–slow6) and compared, within each band, the dominance of gyral versus sulcal frequency characteristics across brain regions. Specifically, if the gyral characteristics in a given frequency band were significantly greater than the sulcal characteristics within a region, that region was considered to be gyri-dominated in that frequency band.

Fig. 3 shows the dominant relationship between gyri and sulci in each brain region after a *t*-test with Bonferroni correction across the six frequency bands. In the PP group, gyri dominated most brain regions in both high- and low-frequency bands; sulci dominated the temporal lobe and left parietal lobe regions in the high-frequency bands (slow1 and slow2), as well as the temporal lobe and left frontal lobe regions in the low-frequency bands (slow5 and slow6). The mid-frequency band slow3 was dominated mainly by gyri, and there was little to no difference between gyral and sulcal frequency characteristics in slow4. In the PT group, the range of gyri-dominated brain regions was reduced, and the range of sulci-dominated brain regions was increased compared with the PP group. In the high-frequency bands (slow1 and slow2), gyral dominance was less extensive (e.g., in the occipital and inferior temporal lobes), while the remaining regions were dominated by sulci. In the mid-frequency band slow3, sulci dominated parts of the frontal and parietal regions, while gyri dominated the rest. From slow4 through the low-frequency band slow6, gyri were mainly dominant, with the occipital lobe and parts of the temporal lobe dominated by sulci. In the TT group, sulcal dominance was observed across most brain regions and frequency bands, with only a few regions remaining gyri-dominated in slow4. In summary, the model-derived gyral and sulcal frequency characteristics showed different spatial dominance patterns across the three neonatal groups and frequency bands, and two general patterns were observed: (1) the spatial extent of sulcal dominance was smallest in the PP group, greater in the PT group, and largest in the TT group; and (2) sulcal dominance was generally more spatially extensive in higher- than lower-frequency bands.

**Fig. 3:**
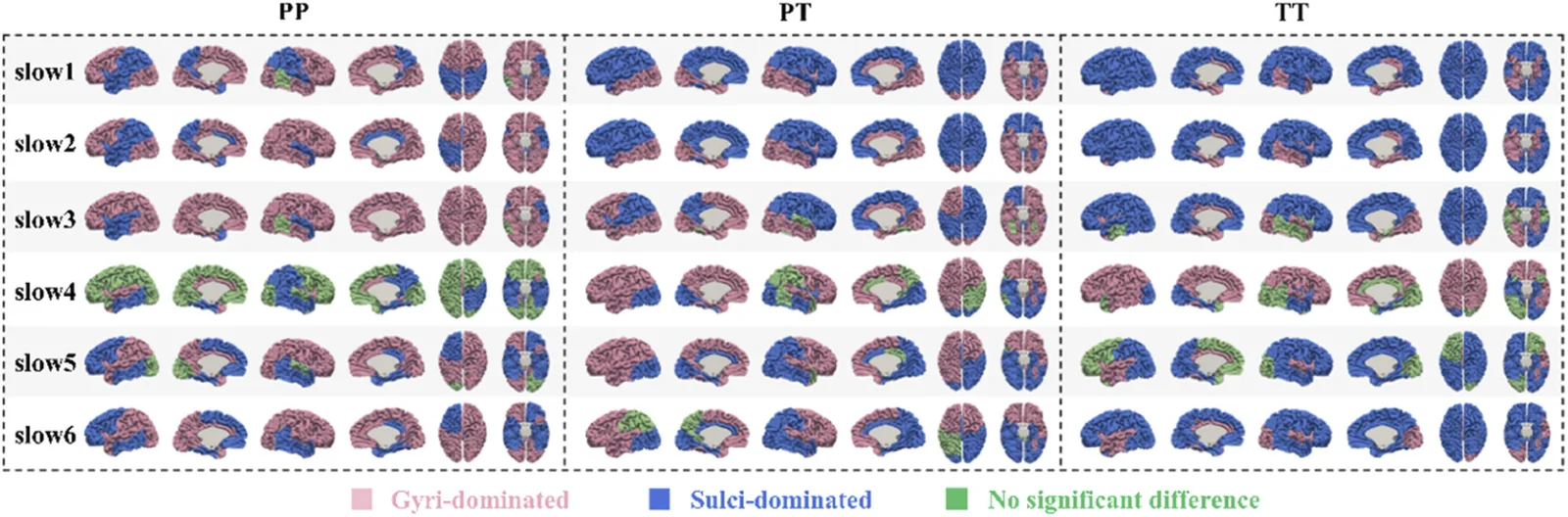
Spatial distribution of band-specific dominance in model-derived gyral and sulcal frequency characteristics across brain regions. PP, preterm-born neonates scanned at preterm age; PT, preterm-born neonates scanned at term-equivalent age; TT, term-born neonates scanned at term age. Pink indicates brain regions dominated by gyri (i.e., gyral characteristics significantly greater than sulcal characteristics), blue indicates regions dominated by sulci, and green indicates regions with no significant differences between gyral and sulcal characteristics.

### 3.3 Pairwise dissimilarities in model-derived frequency characteristics

We calculated the Euclidean distances between the model-derived gyral, sulcal, and gyro-sulcal differentiation sequences to quantify the degree of intergroup dissimilarity in their frequency-characteristic patterns. All six frequency bands were first examined in the band-specific dominance analysis shown in Fig. 3. For focused follow-up comparisons, slow2 was selected as a representative high-frequency band because it showed clear cross-group variation in the spatial distribution of gyral and sulcal dominance. Slow6 was selected as a representative low-frequency band because it represented the lowest-frequency range and also showed clear cross-group spatial variation. We therefore further investigated pairwise dissimilarities in model-derived frequency characteristics across the full band, slow2, and slow6.

#### 3.3.1 Full-band pairwise dissimilarities

Fig. 4 illustrates the full-band pairwise dissimilarities among the three neonatal groups, quantified as Euclidean distances between the model-derived gyro-sulcal differentiation sequences. The largest dissimilarity was observed between the PP and PT groups, primarily in frontal regions; the dissimilarity between the PP and TT groups was smaller, while the PT and TT groups showed the smallest dissimilarity. These results indicate cross-sectional group differences in gyro-sulcal differentiation. The smaller differences between the PT and TT groups indicate greater similarity between these two groups than between the PP and PT groups (Lee et al., 2012). In addition, minimal differences between the PT and TT groups were observed in the left parietal lobe. By contrast, differences in the occipital lobe were relatively small between the PP and PT groups but were evident between both preterm groups and the TT group, indicating that occipital group differences remained evident in comparisons involving the TT group. Further analyses revealed that intergroup dissimilarities in model-derived gyral frequency characteristics were minimal, whereas sulcal characteristics showed more pronounced differences, largely consistent with the patterns observed in gyro-sulcal differentiation. This finding suggests that the observed intergroup differences were more closely reflected in sulcal than gyral frequency characteristics.

**Fig. 4:**
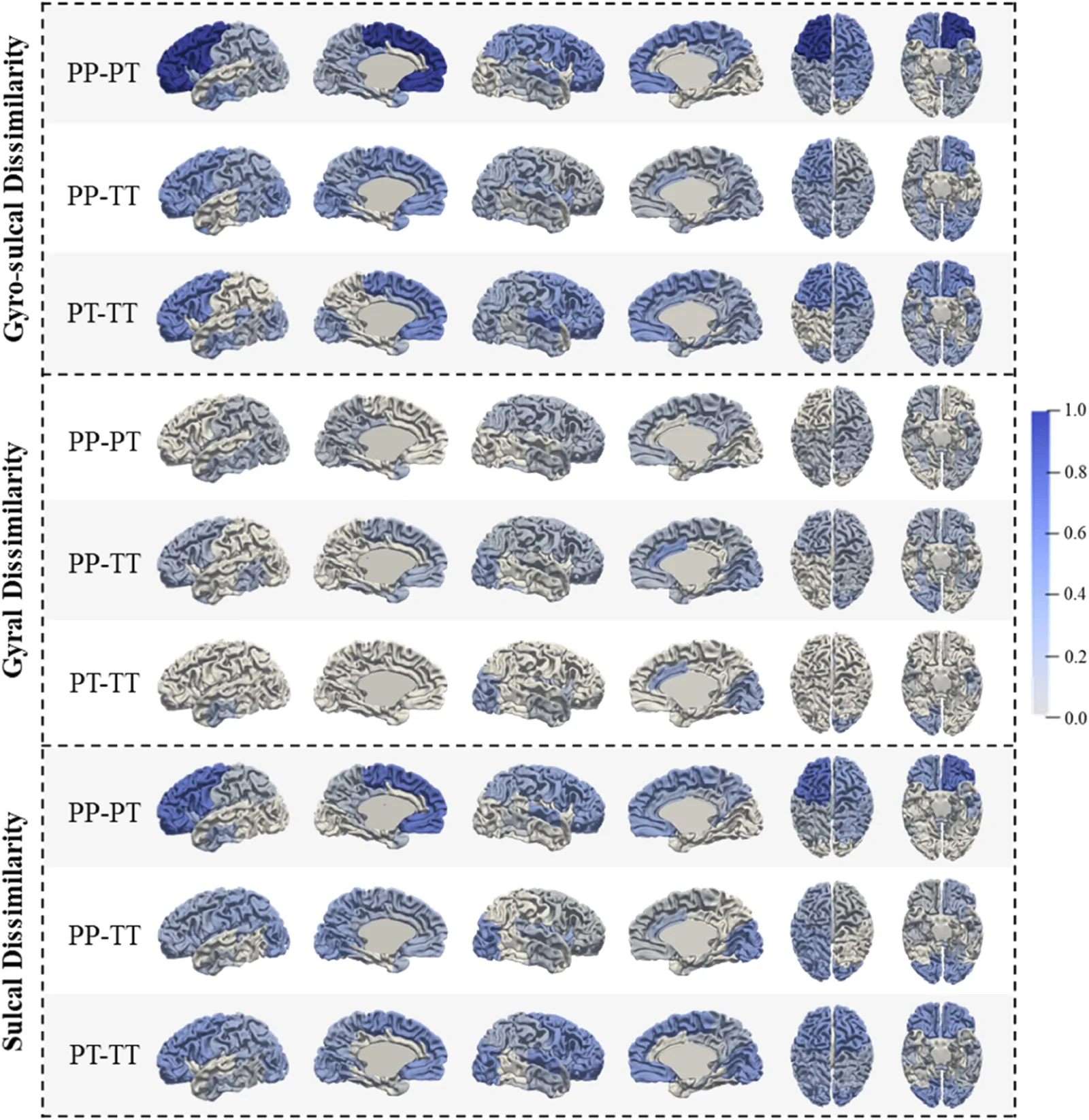
Pairwise dissimilarities in model-derived full-band frequency characteristics among the three neonatal groups. Panels show model-derived gyro-sulcal differentiation characteristics, model-derived gyral frequency characteristics, and model-derived sulcal frequency characteristics. PP–PT, PP–TT, and PT–TT denote the three pairwise group comparisons. PP, preterm-born neonates scanned at preterm age; PT, preterm-born neonates scanned at term-equivalent age; TT, term-born neonates scanned at term age.

#### 3.3.2 High-frequency slow2-band pairwise dissimilarities

Fig. 5 shows the group differences in gyro-sulcal frequency characteristics within the slow2 band. The largest dissimilarities in the model-derived gyro-sulcal differentiation characteristics were observed in the frontal lobe between the PP group and both the PT and TT groups, while the PT and TT groups showed relatively smaller dissimilarities. This pattern is consistent with the full-band findings, indicating cross-sectional group differences in high-frequency gyro-sulcal differentiation. The PT and TT groups showed smaller frontal differences than comparisons involving the PP group. However, Fig. 5 further demonstrates that the preterm–term group still differed from the term–term group across widespread regions, particularly in the lateral inferior temporal and occipital cortices. Gyral characteristics showed relatively small intergroup differences, whereas the spatial pattern of sulcal characteristics more closely resembled that of gyro-sulcal differentiation.

**Fig. 5:**
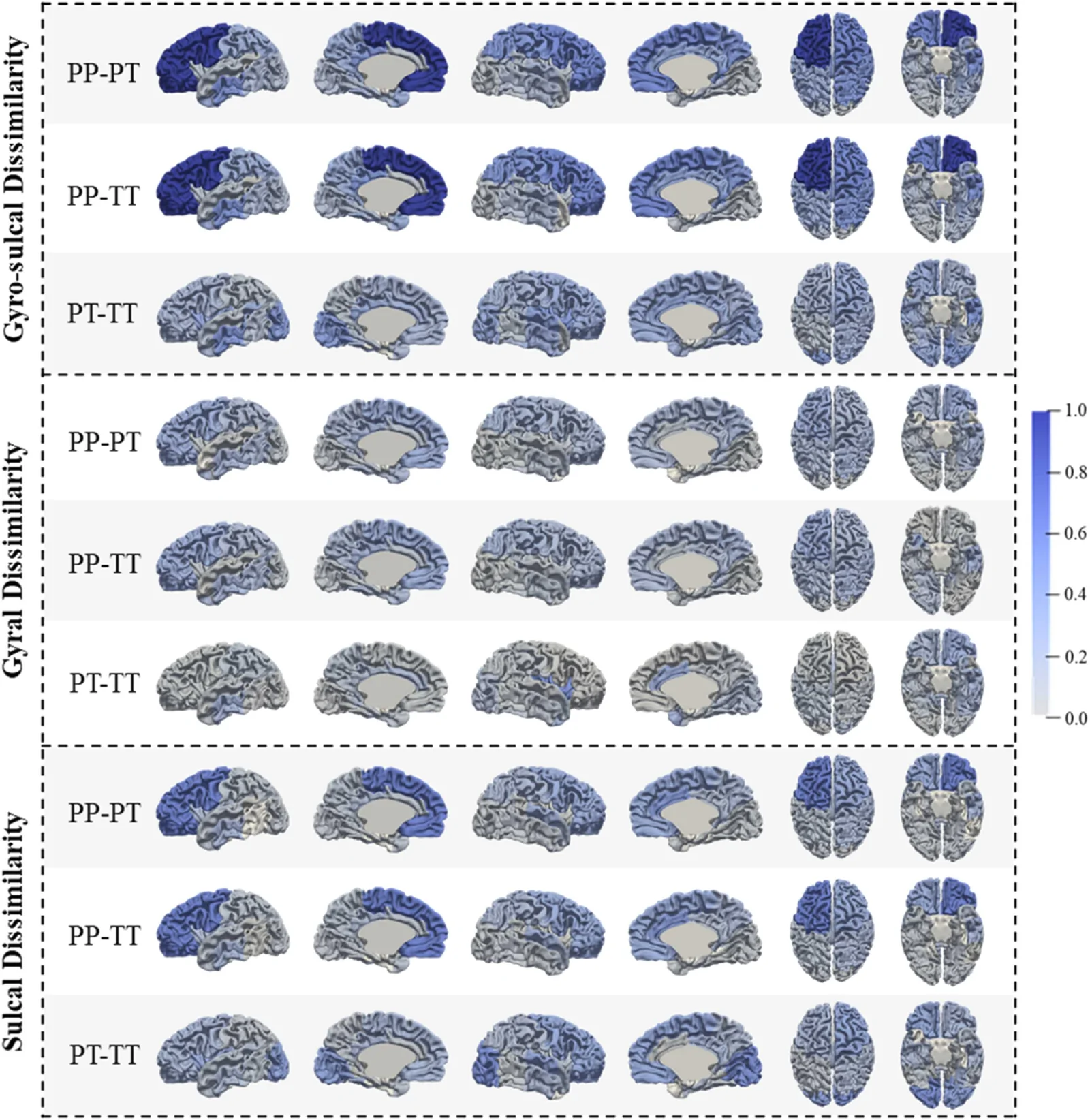
**Pairwise dissimilarities in model-derived slow2-band frequency characteristics among the three neonatal groups.** Panels show model-derived gyro-sulcal differentiation characteristics, model-derived gyral frequency characteristics, and model-derived sulcal frequency characteristics. PP–PT, PP–TT, and PT–TT denote the three pairwise group comparisons. PP, preterm-born neonates scanned at preterm age; PT, preterm-born neonates scanned at term-equivalent age; TT, term-born neonates scanned at term age.

#### 3.3.3 Low-Frequency Slow6-Band Pairwise Dissimilarities

Fig. 6 shows the pairwise dissimilarities in model-derived frequency characteristics among the three neonatal groups in the low-frequency slow6 band. Gyro-sulcal differentiation patterns in the frontal, parietal, and superior lateral temporal lobe regions showed intergroup dissimilarities, but the magnitude of these dissimilarities was smaller than that observed in the high-frequency band. The dissimilarity patterns in the frontal and parietal lobes were more closely reflected in sulcal characteristics, whereas those in the superior temporal lobe were more closely reflected in gyral characteristics. Overall, intergroup dissimilarities in slow6 were smaller than those in slow2 and were more closely reflected in sulcal than gyral frequency characteristics.

**Fig. 6:**
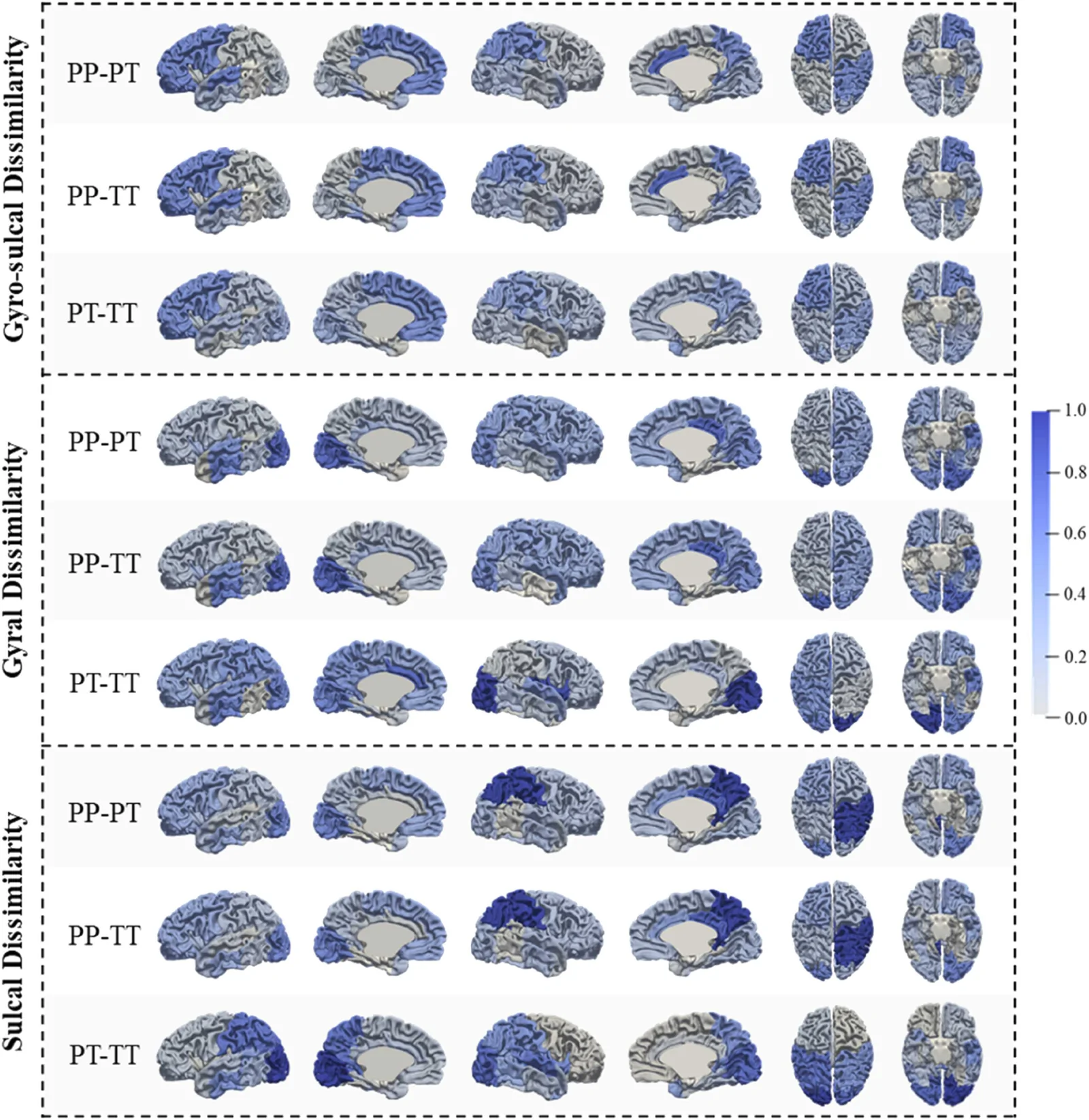
Pairwise dissimilarities in model-derived slow6-band frequency characteristics among the three neonatal groups. Panels show model-derived gyro-sulcal differentiation characteristics, model-derived gyral frequency characteristics, and model-derived sulcal frequency characteristics. PP–PT, PP–TT, and PT–TT denote the three pairwise group comparisons. PP, preterm-born neonates scanned at preterm age; PT, preterm-born neonates scanned at term-equivalent age; TT, term-born neonates scanned at term age.

### 3.4 Gene expression associations with model-derived frequency characteristics

We selected 120 genes associated with cortical morphology to assess the correlations between gene expression and the model-derived sulcal frequency characteristics, gyral frequency characteristics, and gyro-sulcal differentiation characteristics across the full band, the high-frequency band (slow2), and the low-frequency band (slow6). Group-level differences among the three neonatal groups are summarized in Fig. 7. From the full-band perspective, the group-level differences in correlations between gene expression and gyro-sulcal differentiation characteristics were consistent with those observed for the correlations between gene expression and model-derived sulcal frequency characteristics. Specifically, significant differences were identified between the PP and PT groups, as well as between the PP and TT groups, indicating that the major differences in imaging–transcriptomic associations among neonates are primarily reflected in associations between gene expression and model-derived sulcal frequency characteristics. Results from the high-frequency band (slow2) and low-frequency band (slow6) further demonstrated that the overall correlation patterns across the full band more closely resembled those of the high-frequency band, suggesting that intergroup differences in these associations were more closely reflected in high-frequency model-derived characteristics.

**Fig. 7:**
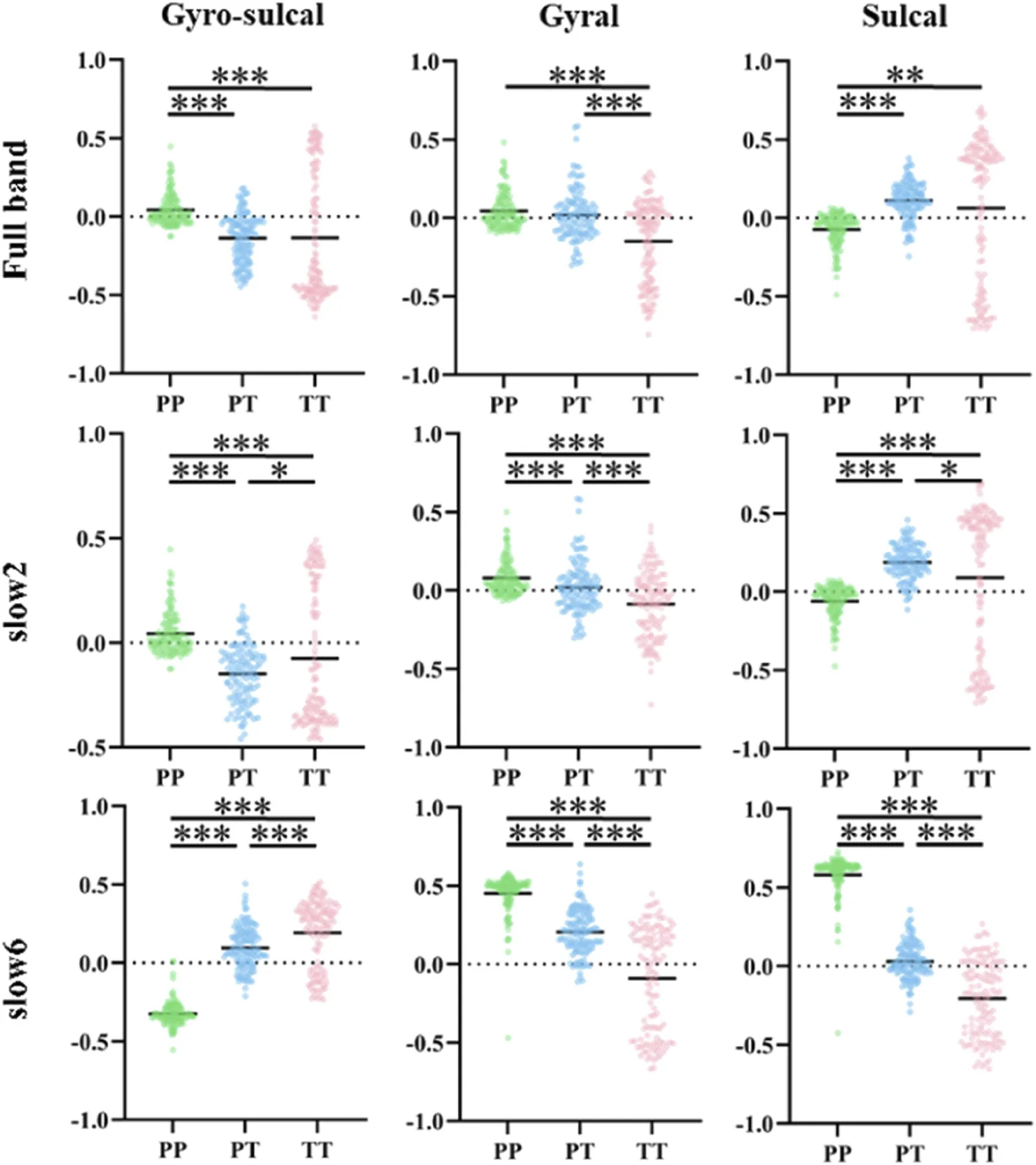
Associations between gene expression and model-derived neonatal brain frequency characteristics. Associations of gene expression with model-derived gyro-sulcal differentiation characteristics, model-derived gyral frequency characteristics, and model-derived sulcal frequency characteristics. The vertical axis represents Pearson’s correlation coefficient r between regional gene-expression patterns and the corresponding model-derived frequency characteristics. Horizontal lines indicate pairwise group comparisons. *p < 0.05, **p < 0.01, and ***p < 0.001. PP–PT, PP–TT, and PT–TT denote the three pairwise group comparisons. PP, preterm-born neonates scanned at preterm age; PT, preterm-born neonates scanned at term-equivalent age; TT, term-born neonates scanned at term age.

## 4 Discussion

This study is one of the first to investigate frequency-specific functional differences between gyri and sulci in the neonatal brain. Using a 1D-CNN model, we performed regional-scale classification of gyral and sulcal BOLD signals across three neonatal groups: preterm neonates scanned immediately after birth, preterm neonates scanned at term-equivalent age, and term neonates. Extensive results reveal three main findings. First, the model showed greater separability between gyral and sulcal BOLD signal patterns in temporal regions than in occipital regions, providing model-derived evidence of regional variation in gyro-sulcal functional differentiation. Second, group differences in the model-derived gyro-sulcal frequency characteristics were observed predominantly in the frontal lobe across both high- and low-frequency bands, and in the temporal and parietal lobes specifically in the high-frequency band. Notably, sulcal dominance was more spatially extensive in the PT and TT groups than in the PP group, particularly in higher-frequency bands. Third, model-derived gyro-sulcal frequency characteristics showed regional associations with cortical gene-expression patterns, indicating spatial correspondence between the imaging and transcriptomic measures.

### 4.1 Regional differences in gyro-sulcal functional differentiation

Our study demonstrated that a deep learning–based model can effectively discriminate between gyral and sulcal BOLD signal patterns across brain regions in neonates. Across the three neonatal groups, the model achieved greater separation of gyral and sulcal BOLD signal patterns in temporal regions than in occipital regions. This regional variation may provide indirect evidence of differences in gyro-sulcal functional organization. This spatial pattern is slightly inconsistent with previous studies in adults reporting higher gyro-sulcal functional differentiation in both the temporal and occipital lobes (Liu et al., 2019, Wang et al., 2024, Zhang et al., 2019). One possible explanation is that during early development, the temporal lobe, which encompasses auditory-related regions, undergoes active gyrification beginning in late gestation, thereby exhibiting relatively mature structural and functional differentiation shortly after birth (Armstrong et al., 1995, Huttenlocher and Dabholkar, 1997). In contrast, the relatively lower degree of gyro–sulcal functional differentiation in the occipital lobe in neonates compared with adults may reflect ongoing cortical maturation. Cortical folding and structural growth follow a tightly regulated developmental timetable that continues after birth, indicating that prenatal cortical development is not yet complete (Namburete et al., 2023, Tau and Peterson, 2010). Moreover, functional connectivity and maturation across cortical regions exhibit a posterior-to-anterior developmental gradient, suggesting that posterior regions, including the occipital lobe, remain under active development at birth (Desrosiers et al., 2024).

### 4.2 Cross-sectional group patterns of sulcal dominance

We further examined the model-derived gyro-sulcal frequency characteristics across the three neonatal groups and identified distinct cross-sectional group patterns. In the PP group, sulcal dominance was relatively limited, with most brain regions dominated by gyral characteristics. In the PT group, sulcal dominance was observed across a broader range of cortical regions than in the PP group. In the TT group, sulcal dominance was distributed across widespread cortical regions. Overall, the spatial extent of sulcal dominance was smallest in the PP group, greater in the PT group, and largest in the TT group. These cross-sectional patterns are broadly consistent with previous studies reporting pronounced cortical remodeling in sulcal regions during early development (Meng et al., 2014, Tung et al., 2026). These cross-sectional findings indicate that the relative contribution of model-derived sulcal frequency characteristics differed across the three neonatal groups and may be relevant to early cortical functional organization (Im, 2021, Tung et al., 2026).

### 4.3 Frequency-band specificity of sulcal dominance

To further characterize the frequency-band specificity of these group patterns, we compared the spatial extent of sulcal dominance across frequency bands. The broader sulcal dominance observed in the PT group than in the PP group was particularly evident in the high-frequency bands, whereas the TT group showed widespread sulcal dominance across both low- and high-frequency bands. Overall, the spatial extent of sulcal dominance was greater in higher- than lower-frequency bands. This model-derived pattern was broadly consistent with prior evidence that sulcal functional signals contain relatively greater high-frequency components, whereas gyral signals are more concentrated in lower-frequency ranges, suggesting that sulci may play more prominent and complex roles in information integration during early development (Ge et al., 2019, Jiang et al., 2021, Liu et al., 2019, Zhang et al., 2019). The full-band dissimilarity patterns in model-derived gyro-sulcal frequency characteristics were largely consistent with those in the high-frequency band. These patterns were more closely reflected in model-derived sulcal than gyral frequency characteristics.

### 4.4 Prematurity-related differences in model-derived frequency characteristics

Comparison of the model-derived frequency characteristics among the three groups revealed that dissimilarities involving the PP group were generally larger, whereas the PT and TT groups showed greater similarity in their frequency-characteristic patterns. Nevertheless, dissimilarities remained between the PT and TT groups. Previous studies have established that the third trimester represents a critical window of rapid brain development, during which the fetal brain undergoes substantial structural and functional remodeling, including accelerated myelination, synaptogenesis, and large-scale network formation (Bouyssi-Kobar et al., 2016, Kostović and Jovanov-Milosević, 2006, Kostović et al., 2019). Although preterm infants demonstrate developmental progress by term-equivalent age, such as dendritic arborization, neurite outgrowth, and increased tissue complexity, their overall maturation remains inferior to that of term-born neonates (Bourgeois et al., 1989, Dimitrova et al., 2021, Wang et al., 2023b). These cross-sectional group patterns may be related to premature exposure to the extrauterine environment and the loss of the supportive intrauterine milieu, thereby disrupting the coordinated maturation of cortical structure and function (Nava et al., 2024, Yue et al., 2019).

In addition, the PT and TT groups showed relatively small dissimilarities in certain regions, such as the parietal lobe. In contrast, dissimilarities between the PT and TT groups remained evident in the frontal, occipital, and parts of the temporal lobes. These findings suggested that the impact of prematurity on cortical development is region-specific. Preterm birth may differentially alter the structural and functional connectivity between gyri and sulci, potentially contributing to the region-specific differences observed in the present study (Hamouda et al., 2026). Furthermore, MRI studies have reported delayed microstructural maturation in cortical areas, including the occipital, temporal, and somatosensory regions, and normative modeling analyses indicate pronounced spatial heterogeneity in cortical developmental deviations among preterm infants (Dimitrova et al., 2020, Kelly et al., 2024, Wang et al., 2023b).

### 4.5 Gene expression associations

We examined the correlations between gyro-sulcal functional signal frequency differentiation characteristics and gene expression profiles associated with cortical morphology across the three neonatal groups. The results showed that between-group differences in gene–frequency-characteristic correlations were most evident for the high-frequency model-derived characteristics. Moreover, the correlation patterns between gene expression and gyro-sulcal functional signal frequency differentiation characteristics were highly consistent between the full frequency band and the high-frequency band. These findings indicate that the observed group differences in gene-expression associations were more closely related to sulcal than gyral model-derived frequency characteristics.

Previous studies have demonstrated that cortical folding is associated with spatially patterned gene-expression programs. For example, modular gene expression patterns in the developing cortex can predict the future locations of gyri and sulci (de Juan Romero et al., 2015). Transcriptomic studies further indicate that even before cortical folding occurs, regions destined to become gyri and sulci already exhibit distinct gene expression profiles, suggesting the presence of an early molecular protomap underlying cortical folding (de Juan Romero and Borrell, 2017, Singh et al., 2024). In addition, previous studies have shown that cortical morphology at birth reflects the spatiotemporal patterns of gene expression, and that the premature termination of gestation caused by preterm birth may significantly disrupt normal cortical developmental trajectories (Ball et al., 2020). Prenatal cortical development also involves regionally heterogeneous synaptogenesis and substantial reorganization of cortical connectivity, processes that may be particularly vulnerable to preterm birth (Huttenlocher and Dabholkar, 1997, Kostović and Jovanov-Milosević, 2006). Therefore, observed associations between gene expression and model-derived frequency characteristics associations observed in this study may reflect regional correspondence between cortical gene expression and model-derived gyro-sulcal frequency-characteristic patterns, although they do not establish a causal molecular mechanism.

### 4.6 Limitations and future work

Several limitations should be acknowledged. First, although the present dataset provides valuable insights into neonatal brain organization, the sample size remains relatively limited, which may reduce statistical power and the generalizability of the findings (Button et al., 2013). Future studies with larger and more diverse cohorts are warranted to validate and extend the present observations (Alex et al., 2024). Second, the current analysis is based on cross-sectional data, which limits our ability to characterize developmental trajectories and to determine whether the observed early-life brain features are associated with later neurodevelopmental or psychiatric outcomes. Longitudinal neuroimaging studies that follow infants across early development and into later childhood will be essential to clarify the long-term functional and clinical significance of these findings (Gui et al., 2019, Liu et al., 2024, Wu et al., 2020). Third, this work primarily relies on a single imaging modality. Integrating multimodal data, such as diffusion MRI, structural MRI, and electrophysiological or molecular measures, may provide a more comprehensive characterization of the relationship between brain structure and function and help uncover the biological mechanisms underlying early cortical development (Jiang et al., 2023a). Fourth, sulcal dominance was defined according to the relative magnitude of model-derived filter-spectrum characteristics and may be influenced by factors other than biological maturation. Cortical geometry may affect the mapping and spatial sampling of fMRI signals on the cortical surface, while residual differences in signal quality, motion-related effects, image registration, interpolation, and surface mapping may also influence the learned frequency characteristics (Ciantar et al., 2022, Fitzgibbon et al., 2020). Although a standardized preprocessing pipeline was applied, potential region-dependent effects of these factors cannot be fully excluded. These factors were not explicitly quantified in the present analysis. Future studies should evaluate the robustness of the observed patterns after controlling for cortical geometric and signal-quality measures and using alternative mapping or preprocessing strategies. Fifth, the current gene-expression analysis focused on regional imaging–transcriptomic associations and did not identify specific genes or pathways. Future gene-level and pathway-enrichment analyses may provide a more detailed biological interpretation of these associations. Sixth, the frequency characteristics examined in this study were derived from learned 1D-CNN filters. Complementary signal-level spectral analyses, repeated model evaluation under different training settings, and validation in independent neonatal datasets would provide additional evidence regarding the robustness and biological relevance of these model-derived patterns.

### 4.7 Conclusion

Despite these limitations, this study revealed potential links among cortical morphological development, model-derived frequency characteristics, and gene-expression patterns. These findings advanced the understanding of neonatal cortical functional organization and prematurity-related differences in neonatal brain organization, thereby providing preliminary evidence for future investigation of neurodevelopmental assessment approaches based on model-derived frequency characteristics. Furthermore, they lay the groundwork for future longitudinal and multimodal studies aimed at elucidating the developmental and clinical significance of early brain connectivity.

## Funding

X.J. was partly supported by 10.13039/501100001809National Natural Science Foundation of China (No. 62276050, No. 62576077), and the Sichuan Science and Technology Program (No. 2024NSFSC0655).

## CRediT authorship contribution statement

**Wei Mao:** Writing – original draft, Visualization, Software, Methodology, Formal analysis, Data curation. **Xun Ou:** Writing – review & editing, Data curation. **Wenxuan Huang:** Writing – review & editing, Visualization, Data curation. **Xuewei Jin:** Writing – review & editing, Data curation. **Keith Kendrick:** Writing – review & editing. **Xi Jiang:** Writing – review & editing, Supervision, Project administration, Funding acquisition, Conceptualization.

## Declaration of Competing Interest

The authors declare that they have no competing interests.

## Data Availability

Data will be made available on request.

## References

[bib1] Alex A.M., Aguate F., Botteron K., Buss C., Chong Y.S., Dager S.R., Donald K.A., Entringer S., Fair D.A., Fortier M.V., Gaab N., Gilmore J.H., Girault J.B., Graham A.M., Groenewold N.A., Hazlett H., Lin W., Meaney M.J., Piven J., Qiu A., Rasmussen J.M., Roos A., Schultz R.T., Skeide M.A., Stein D.J., Styner M., Thompson P.M., Turesky T.K., Wadhwa P.D., Zar H.J., Zöllei L., de Los Campos G., Knickmeyer R.C. (2024). A global multicohort study to map subcortical brain development and cognition in infancy and early childhood. Nat. Neurosci..

[bib2] Armstrong E., Schleicher A., Omran H., Curtis M., Zilles K. (1995). The ontogeny of human gyrification. Cereb. Cortex.

[bib3] Ball G., Seidlitz J., O'Muircheartaigh J., Dimitrova R., Fenchel D., Makropoulos A., Christiaens D., Schuh A., Passerat-Palmbach J., Hutter J., Cordero-Grande L., Hughes E., Price A., Hajnal J.V., Rueckert D., Robinson E.C., Edwards A.D. (2020). Cortical morphology at birth reflects spatiotemporal patterns of gene expression in the fetal human brain. PLoS. Biol..

[bib4] Bitner-Glindzicz M., Rahman S., Chant K., Marlow N. (2014). Gentamicin, genetic variation and deafness in preterm children. BMC Pediatr..

[bib5] Blencowe H., Lawn J.E., Vazquez T., Fielder A., Gilbert C. (2013). Preterm-associated visual impairment and estimates of retinopathy of prematurity at regional and global levels for 2010. Pediatr. Res..

[bib6] Bourgeois J.P., Jastreboff P.J., Rakic P. (1989). Synaptogenesis in visual cortex of normal and preterm monkeys: evidence for intrinsic regulation of synaptic overproduction. Proc. Natl. Acad. Sci..

[bib7] Bouyssi-Kobar M., du Plessis A.J., McCarter R., Brossard-Racine M., Murnick J., Tinkleman L., Robertson R.L., Limperopoulos C. (2016). Third trimester brain growth in preterm infants compared with in utero healthy fetuses. Pediatrics.

[bib8] Button K.S., Ioannidis J.P., Mokrysz C., Nosek B.A., Flint J., Robinson E.S., Munafò M.R. (2013). Power failure: why small sample size undermines the reliability of neuroscience. Nat. Rev. Neurosci..

[bib9] Chawanpaiboon S., Vogel J.P., Moller A.B., Lumbiganon P., Petzold M., Hogan D., Landoulsi S., Jampathong N., Kongwattanakul K., Laopaiboon M., Lewis C., Rattanakanokchai S., Teng D.N., Thinkhamrop J., Watananirun K., Zhang J., Zhou W., Gülmezoglu A.M. (2019). Global, regional, and national estimates of levels of preterm birth in 2014: a systematic review and modelling analysis. Lancet Glob. Health.

[bib10] Chen Y., Yan J., Jiang M., Zhang T., Zhao Z., Zhao W., Zheng J., Yao D., Zhang R., Kendrick K.M., Jiang X. (2024). Adversarial learning based node-edge graph attention networks for autism spectrum disorder identification. IEEE Trans. Neural Netw. Learn. Syst..

[bib11] Ciantar K.G., Farrugia C., Galdi P., Scerri K., Xu T., Bajada C.J. (2022). Geometric effects of volume-to-surface mapping of fMRI data. Brain Struct. Funct..

[bib12] de Juan Romero C., Borrell V. (2017). Genetic maps and patterns of cerebral cortex folding. Curr. Opin. Cell. Biol..

[bib13] de Juan Romero C., Bruder C., Tomasello U., Sanz-Anquela J.M., Borrell V. (2015). Discrete domains of gene expression in germinal layers distinguish the development of gyrencephaly. Embo J..

[bib14] Deng F., Jiang X., Zhu D., Zhang T., Li K., Guo L., Liu T. (2014). A functional model of cortical gyri and sulci. Brain Struct. Funct..

[bib15] Desrosiers J., Caron-Desrochers L., René A., Gaudet I., Pincivy A., Paquette N., Gallagher A. (2024). Functional connectivity development in the prenatal and neonatal stages measured by functional magnetic resonance imaging: a systematic review. Neurosci. Biobehav. Rev..

[bib16] Dimitrova R., Pietsch M., Christiaens D., Ciarrusta J., Wolfers T., Batalle D., Hughes E., Hutter J., Cordero-Grande L., Price A.N., Chew A., Falconer S., Vecchiato K., Steinweg J.K., Carney O., Rutherford M.A., Tournier J.D., Counsell S.J., Marquand A.F., Rueckert D., Hajnal J.V., McAlonan G., Edwards A.D., O'Muircheartaigh J. (2020). Heterogeneity in brain microstructural development following preterm birth. Cereb. Cortex.

[bib17] Dimitrova R., Pietsch M., Ciarrusta J., Fitzgibbon S.P., Williams L.Z.J., Christiaens D., Cordero-Grande L., Batalle D., Makropoulos A., Schuh A., Price A.N., Hutter J., Teixeira R.P., Hughes E., Chew A., Falconer S., Carney O., Egloff A., Tournier J.D., McAlonan G., Rutherford M.A., Counsell S.J., Robinson E.C., Hajnal J.V., Rueckert D., Edwards A.D., O'Muircheartaigh J. (2021). Preterm birth alters the development of cortical microstructure and morphology at term-equivalent age. Neuroimage.

[bib18] Drljan Demeši, Mikov A., Filipović K., Tomašević-Todorović S., Knežević A., Krasnik R. (2016). Cerebral palsy in preterm infants. Vojnosanit. Pregl..

[bib19] Erbek S., Gokmen Z., Ozkiraz S., Erbek S.S., Tarcan A., Ozluoglu L.N. (2009). Vestibular evoked myogenic potentials in preterm infants. Audiol. Neurootol..

[bib20] Eyre M., Fitzgibbon S.P., Ciarrusta J., Cordero-Grande L., Price A.N., Poppe T., Schuh A., Hughes E., O’Keeffe C., Brandon J., Cromb D., Vecchiato K., Andersson J., Duff E.P., Counsell S.J., Smith S.M., Rueckert D., Hajnal J.V., Arichi T., O’Muircheartaigh J., Batalle D., Edwards A.D. (2021). The Developing Human Connectome Project: typical and disrupted perinatal functional connectivity. Brain.

[bib21] Fitzgibbon S.P., Harrison S.J., Jenkinson M., Baxter L., Robinson E.C., Bastiani M., Bozek J., Karolis V., Cordero Grande L., Price A.N., Hughes E., Makropoulos A., Passerat-Palmbach J., Schuh A., Gao J., Farahibozorg S.R., O'Muircheartaigh J., Ciarrusta J., O'Keeffe C., Brandon J., Arichi T., Rueckert D., Hajnal J.V., Edwards A.D., Smith S.M., Duff E., Andersson J. (2020). The developing human connectome project (dHCP) automated resting-state functional processing framework for newborn infants. Neuroimage.

[bib22] França L.G.S, Ciarrusta J., Gale-Grant O., Fenn-Moltu S., Fitzgibbon S., Chew A., Falconer S., Dimitrova R., Cordero-Grande L., Price A.N., Hughes E., O’Muircheartaigh J., Duff E., Tuulari J.J., Deco G., Counsell S.J., Hajnal J.V., Nosarti C., Arichi T., Edwards A.D., McAlonan G., Batalle D. (2024). Neonatal brain dynamic functional connectivity in term and preterm infants and its association with early childhood neurodevelopment. Nat. Commun..

[bib23] Franz A.P., Bolat G.U., Bolat H., Matijasevich A., Santos I.S., Silveira R.C., Procianoy R.S., Rohde L.A., Moreira-Maia C.R. (2018). Attention-Deficit/Hyperactivity Disorder and Very Preterm/Very Low Birth Weight: A Meta-analysis. Pediatrics.

[bib24] Ge, F., Zhang, S., Huang, H., Jiang, X., Dong, Q., Guo, L., Wang, X., Liu, T., 2019. Exploring Intrinsic Functional Differences of Gyri, Sulci and 2-Hinge, 3-Hinge Joints on Cerebral Cortex. 2019 IEEE 16th International Symposium on Biomedical Imaging (ISBI 2019), pp. 1585-1589.

[bib25] Gousias I.S., Edwards A.D., Rutherford M.A., Counsell S.J., Hajnal J.V., Rueckert D., Hammers A. (2012). Magnetic resonance imaging of the newborn brain: manual segmentation of labelled atlases in term-born and preterm infants. Neuroimage.

[bib26] Gui L., Loukas S., Lazeyras F., Hüppi P.S., Meskaldji D.E., Borradori Tolsa C. (2019). Longitudinal study of neonatal brain tissue volumes in preterm infants and their ability to predict neurodevelopmental outcome. Neuroimage.

[bib27] Hagan A.T., Xu L., Kou J., Hu Y., Klugah-Brown B., Li J., Bore M.C., Becker B., Kendrick K.M., Jiang X. (2025). Oxytocin modulation of resting-state functional connectivity network topology in individuals with higher autistic traits. Psychoradiology.

[bib28] Hamouda E., Mao W., Xu D., Kendrick K., Jiang X. (2026). Preterm birth differentially impacts structural and functional connectivity of cortical gyri and sulci. Dev. Cogn. Neurosci..

[bib29] Hedderich D.M, Bäuml J.G., Berndt M.T., Menegaux A., Scheef L., Daamen M., Zimmer C., Bartmann P., Boecker H., Wolke D., Gaser C., Sorg C. (2019). Aberrant gyrification contributes to the link between gestational age and adult IQ after premature birth. Brain.

[bib30] Hee Chung E., Chou J., Brown K.A. (2020). Neurodevelopmental outcomes of preterm infants: a recent literature review. Transl. Pediatr..

[bib31] Hilgetag C.C., Barbas H. (2005). Developmental mechanics of the primate cerebral cortex. Anat. Embryol..

[bib32] Huttenlocher P.R., Dabholkar A.S. (1997). Regional differences in synaptogenesis in human cerebral cortex. J. Comp. Neurol..

[bib33] Im K., Martin C.R., Preedy V.R., Rajendram R. (2021). Factors Affecting Neurodevelopment.

[bib34] Jenkinson M., Bannister P., Brady M., Smith S. (2002). Improved optimization for the robust and accurate linear registration and motion correction of brain images. Neuroimage.

[bib35] Jenkinson M., Smith S. (2001). A global optimisation method for robust affine registration of brain images. Med. Image Anal..

[bib36] Jiang M., Chen Y., Yan J., Xiao Z., Mao W., Zhao B., Yang S., Zhao Z., Zhang T., Guo L., Becker B., Yao D., Kendrick K.M., Jiang X. (2024). Anatomy-guided spatio-temporal graph convolutional networks (AG-STGCNs) for modeling functional connectivity between gyri and sulci across multiple task domains. IEEE Trans. Neural Netw. Learn. Syst..

[bib37] Jiang X., Li X., Lv J., Zhang T., Zhang S., Guo L., Liu T. (2015). Sparse representation of HCP grayordinate data reveals novel functional architecture of cerebral cortex. Hum. Brain Mapp..

[bib38] Jiang X., Li X., Lv J., Zhao S., Zhang S., Zhang W., Zhang T., Han J., Guo L., Liu T. (2018). Temporal dynamics assessment of spatial overlap pattern of functional brain networks reveals novel functional architecture of cerebral cortex. IEEE Trans. Biomed. Eng..

[bib39] Jiang X., Shou X.J., Zhao Z., Chen Y., Meng F.C., Le J., Song T.J., Xu X.J., Guo W., Ke X., Cai X.E., Zhao W., Kou J., Huo R., Liu Y., Yuan H.S., Xing Y., Han J.S., Han S.P., Li Y., Lai H., Zhang L., Jia M.X., Liu J., Liu X., Kendrick K.M., Zhang R. (2023). A brain structural connectivity biomarker for autism spectrum disorder diagnosis in early childhood. Psychoradiology.

[bib40] Jiang X., Yan J., Zhao Y., Jiang M., Chen Y., Zhou J., Xiao Z., Wang Z., Zhang R., Becker B., Zhu D., Kendrick K.M., Liu T. (2023). Characterizing functional brain networks via Spatio-Temporal Attention 4D Convolutional Neural Networks (STA-4DCNNs). Neural Netw..

[bib41] Jiang X., Zhang T., Zhang S., Kendrick K.M., Liu T. (2021). Fundamental functional differences between gyri and sulci: implications for brain function, cognition, and behavior. Psychoradiology.

[bib42] Kelly C.E., Thompson D.K., Adamson C.L., Ball G., Dhollander T., Beare R., Matthews L.G., Alexander B., Cheong J.L.Y., Doyle L.W., Anderson P.J., Inder T.E. (2024). Cortical growth from infancy to adolescence in preterm and term-born children. Brain.

[bib43] Kostović I., Jovanov-Milosević N. (2006). The development of cerebral connections during the first 20-45 weeks' gestation. Semin. Fetal Neonatal Med..

[bib44] Kostović I., Sedmak G., Judaš M. (2019). Neural histology and neurogenesis of the human fetal and infant brain. Neuroimage.

[bib45] Kuzniewicz M.W., Wi S., Qian Y., Walsh E.M., Armstrong M.A., Croen L.A. (2014). Prevalence and neonatal factors associated with autism spectrum disorders in preterm infants. J. Pediatr..

[bib46] Lee W., Donner E.J., Nossin-Manor R., Whyte H.E., Sled J.G., Taylor M.J. (2012). Visual functional magnetic resonance imaging of preterm infants. Dev. Med. Child. Neurol..

[bib47] Li M., Santpere G., Imamura Kawasawa Y., Evgrafov O.V., Gulden F.O., Pochareddy S., Sunkin S.M., Li Z., Shin Y., Zhu Y., Sousa A.M.M., Werling D.M., Kitchen R.R., Kang H.J., Pletikos M., Choi J., Muchnik S., Xu X., Wang D., Lorente-Galdos B., Liu S., Giusti-Rodríguez P., Won H., de Leeuw C.A., Pardiñas A.F., Hu M., Jin F., Li Y., Owen M.J., O'Donovan M.C., Walters J.T.R., Posthuma D., Reimers M.A., Levitt P., Weinberger D.R., Hyde T.M., Kleinman J.E., Geschwind D.H., Hawrylycz M.J., State M.W., Sanders S.J., Sullivan P.F., Gerstein M.B., Lein E.S., Knowles J.A., Sestan N. (2018). Integrative functional genomic analysis of human brain development and neuropsychiatric risks. Science.

[bib48] Lima G.M., Marba S.T., Santos M.F. (2006). Hearing screening in a neonatal intensive care unit. J. Pediatr..

[bib49] Liu H., Jiang X., Zhang T., Ren Y., Hu X., Guo L., Han J., Liu T. (2017). Elucidating functional differences between cortical gyri and sulci via sparse representation HCP grayordinate fMRI data. Brain Res..

[bib50] Liu H., Zhang S., Jiang X., Zhang T., Huang H., Ge F., Zhao L., Li X., Hu X., Han J., Guo L., Liu T. (2019). The cerebral cortex is bisectionally segregated into two fundamentally different functional units of Gyri and Sulci. Cereb. Cortex.

[bib51] Liu L., Oza S., Hogan D., Chu Y., Perin J., Zhu J., Lawn J.E., Cousens S., Mathers C., Black R.E. (2016). Global, regional, and national causes of under-5 mortality in 2000-15: an updated systematic analysis with implications for the Sustainable Development Goals. Lancet.

[bib52] Liu M., Lu M., Kim S.Y., Lee H.J., Duffy B.A., Yuan S., Chai Y., Cole J.H., Wu X., Toga A.W., Jahanshad N., Gano D., Barkovich A.J., Xu D., Kim H. (2024). Brain age predicted using graph convolutional neural network explains neurodevelopmental trajectory in preterm neonates. Eur. Radiol..

[bib53] Makropoulos A., Robinson E.C., Schuh A., Wright R., Fitzgibbon S., Bozek J., Counsell S.J., Steinweg J., Vecchiato K., Passerat-Palmbach J., Lenz G., Mortari F., Tenev T., Duff E.P., Bastiani M., Cordero-Grande L., Hughes E., Tusor N., Tournier J.D., Hutter J., Price A.N., Teixeira R., Murgasova M., Victor S., Kelly C., Rutherford M.A., Smith S.M., Edwards A.D., Hajnal J.V., Jenkinson M., Rueckert D. (2018). The developing human connectome project: A minimal processing pipeline for neonatal cortical surface reconstruction. Neuroimage.

[bib54] Mao W., He Z., Jin X., Hamouda E., Ou X., Kendrick K.M., Zhang T., Jiang X. (2026). Structuro‐Functional Differentiation and Coupling of Gyri and Sulci in the Neonatal Cortex. Hum. Brain Mapp..

[bib55] Meng Y., Li G., Lin W., Gilmore J.H., Shen D. (2014). Spatial distribution and longitudinal development of deep cortical sulcal landmarks in infants. Neuroimage.

[bib56] Moreno-Plata H., Rangel-Nava H.A., Félix-Arce C., Valdovinos-Chávez S., Méndez Lozano D.H. (2011). [Short-term morbidity in newborns of the late preterm period]. Ginecol. Obstet. Mex..

[bib57] Mwaniki M.K., Atieno M., Lawn J.E., Newton C.R. (2012). Long-term neurodevelopmental outcomes after intrauterine and neonatal insults: a systematic review. Lancet.

[bib58] Namburete A.I.L., Papież B.W., Fernandes M., Wyburd M.K., Hesse L.S., Moser F.A., Ismail L.C., Gunier R.B., Squier W., Ohuma E.O., Carvalho M., Jaffer Y., Gravett M., Wu Q., Lambert A., Winsey A., Restrepo-Méndez M.C., Bertino E., Purwar M., Barros F.C., Stein A., Noble J.A., Molnár Z., Jenkinson M., Bhutta Z.A., Papageorghiou A.T., Villar J., Kennedy S.H. (2023). Normative spatiotemporal fetal brain maturation with satisfactory development at 2 years. Nature.

[bib59] Nava C., Lupo E., Lista G. (2024). Preterm nutrition and brain development. BMJ Nutr. Prev. & Health.

[bib60] Nie J., Guo L., Li K., Wang Y., Chen G., Li L., Chen H., Deng F., Jiang X., Zhang T., Huang L., Faraco C., Zhang D., Guo C., Yap P.T., Hu X., Li G., Lv J., Yuan Y., Zhu D., Han J., Sabatinelli D., Zhao Q., Miller L.S., Xu B., Shen P., Platt S., Shen D., Hu X., Liu T. (2012). Axonal fiber terminations concentrate on gyri. Cereb. Cortex.

[bib61] Platt M.J. (2014). Outcomes in preterm infants. Public. Health.

[bib62] Rakic P. (2009). Evolution of the neocortex: a perspective from developmental biology. Nat. Rev. Neurosci..

[bib63] Saigal S., Doyle L.W. (2008). An overview of mortality and sequelae of preterm birth from infancy to adulthood. Lancet.

[bib64] Shen D., Wu G., Suk H.I. (2017). Deep Learning in Medical Image Analysis. Annu. Rev. Biomed. Eng..

[bib65] Singh A., Del-Valle-Anton L., de Juan Romero C., Zhang Z., Ortuño E.F., Mahesh A., Espinós A., Soler R., Cárdenas A., Fernández V., Lusby R., Tiwari V.K., Borrell V. (2024). Gene regulatory landscape of cerebral cortex folding. Sci. Adv..

[bib66] Tau G.Z., Peterson B.S. (2010). Normal development of brain circuits. Neuropsychopharmacology.

[bib67] Tung S.S., Yan X., Fascendini B., Tyagi C., Reyes C.M., Ducre K., Perez K., Allen A., Horenziak J., Wu H., Keil B., Natu V.S., Grill-Spector K. (2026). How do infant brains fold? Sulcal deepening is linked to development of sulcal span, thickness, curvature, and microstructure. Commun. Biol..

[bib68] Wang L., Yang Y., Hu X., Zhao S., Jiang X., Guo L., Han J., Liu T. (2024). Frequency-specific functional difference between gyri and sulci in naturalistic paradigm fMRI. Brain Struct. Funct..

[bib69] Wang Q., Zhao S., He Z., Zhang S., Jiang X., Zhang T., Liu T., Liu C., Han J. (2023). Modeling functional difference between gyri and sulci within intrinsic connectivity networks. Cereb. Cortex.

[bib70] Wang W., Yu Q., Liang W., Xu F., Li Z., Tang Y., Liu S. (2023). Altered cortical microstructure in preterm infants at term-equivalent age relative to term-born neonates. Cereb. Cortex.

[bib71] Wu D., Chang L., Ernst T.M., Caffo B.S., Oishi K. (2020). Developmental score of the infant brain: characterizing diffusion MRI in term- and preterm-born infants. Brain Struct. Funct..

[bib72] Yan J., Chen Y., Xiao Z., Zhang S., Jiang M., Wang T., Zhang T., Lv J., Becker B., Zhang R., Zhu D., Han J., Yao D., Kendrick K.M., Liu T., Jiang X. (2022). Modeling spatio-temporal patterns of holistic functional brain networks via multi-head guided attention graph neural networks (Multi-Head GAGNNs). Med. Image Anal..

[bib73] Yang S., Zhao Z., Cui H., Zhang T., Zhao L., He Z., Liu H., Guo L., Liu T., Becker B., Kendrick K.M., Jiang X. (2019). Temporal Variability of Cortical Gyral-Sulcal Resting State Functional Activity Correlates With Fluid Intelligence. Front. Neural Circuits.

[bib74] Yue S.T., Zhang J., Ma D.H. (2019). Research advances in the effect of environmental stress in the neonatal intensive care unit on the neurodevelopment of preterm infants and its epigenetics]. Zhongguo Dang Dai Er Ke Za Zhi.

[bib75] Zhang S., Liu H., Huang H., Zhao Y., Jiang X., Bowers B., Guo L., Hu X., Sanchez M., Liu T. (2019). Deep learning models unveiled functional difference between cortical Gyri and Sulci. IEEE Trans. Biomed. Eng..

[bib76] Zhao L. (2025). Advances in functional magnetic resonance imaging-based brain function mapping: a deep learning perspective. Psychoradiology.

